# Native T1 mapping detects both acute clinical rejection and graft dysfunction in pediatric heart transplant patients

**DOI:** 10.1186/s12968-022-00875-z

**Published:** 2022-10-03

**Authors:** Devika P. Richmann, Nyshidha Gurijala, Jason G. Mandell, Ashish Doshi, Karin Hamman, Christopher Rossi, Avi Z. Rosenberg, Russell Cross, Joshua Kanter, John T. Berger, Laura Olivieri

**Affiliations:** 1 Children’s National Hospital, Washington, D.C. USA; 2 George Washington University School of Medicine, Washington, D.C. USA; 3 University of Rochester Medical Center, Rochester, NY USA; 4 Johns Hopkins University Children’s Center, Baltimore, MD USA; 5 Johns Hopkins University, Baltimore, MD USA

**Keywords:** Cardiac magnetic resonance, Parametric mapping, Graft rejection, Graft dysfunction, Pediatric heart transplant

## Abstract

**Background:**

Cardiovascular magnetic resonance (CMR) is emerging as an important tool for cardiac allograft assessment. Native T1 mapping may add value in identifying rejection and in assessing graft dysfunction and myocardial fibrosis burden. We hypothesized that CMR native T1 values and features of textural analysis of T1 maps would identify acute rejection, and in a secondary analysis, correlate with markers of graft dysfunction, and with fibrosis percentage from endomyocardial biopsy (EMB).

**Methods:**

Fifty cases with simultaneous EMB, right heart catheterization, and 1.5 T CMR with breath-held T1 mapping via modified Look-Locker inversion recovery (MOLLI) in 8 short-axis slices and subsequent quantification of mean and peak native T1 values, were performed on 24 pediatric subjects. A single mid-ventricular slice was used for image texture analysis using nine gray-level co-occurrence matrix features. Digital quantification of Masson trichrome stained EMB samples established degree of fibrosis. Markers of graft dysfunction, including serum brain natriuretic peptide levels and hemodynamic measurements from echocardiography, catheterization, and CMR were collated. Subjects were divided into three groups based on degree of rejection: acute rejection requiring new therapy, mild rejection requiring increased ongoing therapy, and no rejection with no change in treatment. Statistical analysis included student’s t-test and linear regression.

**Results:**

Peak and mean T1 values were significantly associated with acute rejection, with a monotonic trend observed with increased grade of rejection. Texture analysis demonstrated greater spatial heterogeneity in T1 values, as demonstrated by energy, entropy, and variance, in cases requiring treatment. Interestingly, 2 subjects who required increased therapy despite low grade EMB results had abnormal peak T1 values. Peak T1 values also correlated with increased BNP, right-sided filling pressures, and capillary wedge pressures. There was no difference in histopathological fibrosis percentage among the 3 groups; histopathological fibrosis did not correlate with T1 values or markers of graft dysfunction.

**Conclusion:**

In pediatric heart transplant patients, native T1 values identify acute rejection requiring treatment and may identify graft dysfunction. CMR shows promise as an important tool for evaluation of cardiac grafts in children, with T1 imaging outperforming biopsy findings in the assessment of rejection.

**Supplementary Information:**

The online version contains supplementary material available at 10.1186/s12968-022-00875-z.

## Introduction

Heart transplant remains a life-saving intervention for pediatric patients with advanced heart failure. Despite significant advancements in immunosuppressive therapy, cardiac allograft rejection remains an important cause of mortality and morbidity after transplantation [1, 2]. Allograft rejection and graft failure are often clinically insidious, yet early detection significantly improves outcomes [3]. Identification of allograft rejection is complex; the current gold standard, direct pathologic evaluation of the myocardium via endomyocardial biopsy (EMB), frequently results in false negatives [4–6] due to random sampling of the accessible regions of the right ventricle [7]. Further, EMB requires an invasive procedure for tissue procurement, which while generally safe, involves a risk of cardiac perforation, arrhythmia, effusion, and tricuspid valve injury [5, 8, 9]. Other noninvasive methods of identifying rejection, including echocardiography [10, 11], serum markers [12], and clinical signs and symptoms also carry limitations in detecting rejection but have been used for diagnosis of late graft dysfunction. Characteristics of late graft dysfunction can be diagnosed by hemodynamic measurements through echocardiography and cardiac catheterization [13, 14], and myocardial fibrosis noted on histological evaluation [15].

Cardiovascular magnetic resonance (CMR) offers a diagnostic advantage in detection of myocardial pathology due to its ability to characterize the entire myocardium for evidence of fibrosis or edema using quantitative T1 and T2 weighted techniques [16]. Native T1 values increase with processes that result in myocardial edema and fibrosis [17]. In adult heart transplant recipients, T1 and T2 mapping techniques have been shown to be more sensitive for the detection of acute allograft rejection than EMB, with excellent negative predictive capacity [18–21].

T1 and T2 mapping techniques are complex pulse sequences which require systematic use and careful interpretation to yield accurate data, particularly in the pediatric population [22]. Typical spatial and temporal resolution values favor larger hearts with slower heart rates to minimize cardiac motion artifacts and provide enough time for T1 recovery [23], making the application of parametric mapping in pediatric populations challenging. However, the use of parametric mapping has been successfully demonstrated in other conditions in the pediatric population [22], including myocarditis [24], hypertrophic cardiomyopathy [25], Duchenne muscular dystrophy myocardial disease [26], iron deposition cardiomyopathy [27], and anthracycline cardiotoxicity [28].

Another frequent challenge of using CMR parametric mapping is the variability of native T1 values, in particular between different field strengths [29], vendors, and individual scanners [16]. This is compounded by lack of normative pediatric T1 data to understand and identify disease states. For this reason, there is a consensus to use institutional reference ranges [16, 17], making comparability of measurements across patients and institutions challenging [23]. We hypothesized that in addition to changes in native T1 value, T1 maps from patients with rejection would demonstrate changes in spatial variability of T1 voxel intensities. Texture analysis is a computational imaging analysis method that quantifies spatial heterogeneity using the *relative* intensity differences between neighboring voxels [30, 31], independent of the actual voxel intensities. Texture analysis thus overcomes the lack of normative values available and has been demonstrated to have utility in other conditions, including myocarditis [32, 33], dilated cardiomyopathy [34], and hypertrophic cardiomyopathy [35].

There is a clear need for a non-invasive and more accurate method of detecting acute allograft rejection and late graft dysfunction in pediatric heart transplant patients. The overall aim of this study was to determine the utility of CMR native T1 values in (1) identifying acute graft rejection and (2) assessing graft dysfunction in pediatric heart transplant patients. In secondary analysis, we aimed to compare textural features of parametric maps in cases of rejection versus those without rejection and to assess the relationship of native T1 mapping and histological myocardial fibrosis burden.

## Methods

### Clinical procedure

In this IRB-approved study, with consent/assent as appropriate, heart transplant patients referred for clinically-indicated EMB were prospectively enrolled to undergo noncontrast-CMR at 1.5 T (MAGNETOM Aera, Siemens Healthineers, Erlangen, Germany) followed by cardiac catheterization, with EMB in an adjoining biplane fluoroscopy suite. CMR included right heart catheterization, standard volumetry cines, phase contrast imaging, and breath-held native T1 mapping using Modified Look-Locker Inversion recovery (MOLLI) in eight short-axis slices. The following parameters were used for MOLLI T1 map acquisition: field of view 360 mm × 307 mm, percent phase field of view 50–80% based on patient’s body habitus, and matrix size 1.4 × 1.4 mm. For patients with a RR interval > 700, echo time (TE) was 1.12 ms, repetition time (TR) was 2.80 ms, and flip angle of 35 degrees; for patients with RR interval < 700, TE was 1.06 ms, TR was 1.93 ms, and flip angle of 70 degrees. Slice thickness and slice skip also varied based on patient’s size, ranging from 4–8 mm to 0–2 mm respectively. Measurements of volume, function, and cardiac output were performed using standard offline software (Medis Medical Imaging, AJ Leiden, Netherlands). Catheterization included collection of standard oximetry and hemodynamic data and EMB. If clinically indicated coronary angiography was performed according to surveillance standard.

Clinical data were recorded, including patient demographics, transplant history, rejection history, and serum brain natriuretic peptide (BNP) levels. Hemodynamic measurements from echocardiography (left ventricular (LV) ejection fraction, mitral E/e’), catheterization (right atrial mean pressure, right ventricle (RV) systolic pressure, RV end diastolic pressure (RVEDP), main pulmonary artery mean pressure, and pulmonary capillary wedge pressure), and CMR (LV and RV end diastolic volume) were also included. Serum BNP and hemodynamic measurements listed above were considered markers of graft dysfunction for the purpose of analysis.

### Rejection analysis

Evidence of cellular rejection and antibody mediated rejection on EMB were graded based on the International Society of Heart and Lung Transplantation (ISHLT) guidelines [36] by a surgical pathologist following standard clinical practices. Clinically, rejection was defined based on treatment plan created by transplant team following the catheterization/EMB procedure. Of note, CMR T1 measurements and analyses did not affect treatment decision, as these are not reported with standard clinical data at our institution. Per our institutional protocol, those cases with Grade 2R or above acute cellular rejection on biopsy received new rejection treatment including intravenous steroids, thymoglobulin, immunoglobulins, etc.. Those cases with Grade 0R or 1R acute cellular rejection on biopsy but no hemodynamic compromise did not receive any treatment or modification to immunosuppressive therapy. Cases with Grade 0R or 1R rejection, with hemodynamic compromise, on echocardiogram, catheterization, or CMR, received treatment per transplant team using other data including biopsy, history of rejection, clinical symptoms, and donor specific antibody (DSA) results, without impact of CMR T1 mapping data. Cases were divided into one of three outcome groups (A, B, C), based on their degree of rejection therapy recommended by their physicians, who were blinded to T1 mapping data. Group A included cases with no rejection and thus no changes made to their treatment regimen. Group B included cases with some evidence of rejection which required augmentation of maintenance treatment regimen or initiation of oral steroids under pulse dosing. Group C included cases with significant evidence of acute rejection, who received new rejection treatment such as intravenous (IV) or oral steroids at pulse dosing, thymoglobulin, or intravenous immunoglobulin (IVIG).

### Parametric map analysis

**Fig. 1:**
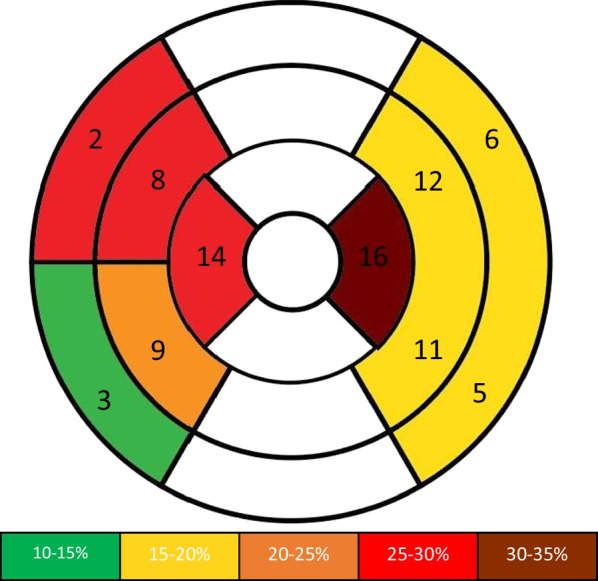
Location representation of regions of interest (ROIs) based on the American Heart Association 17-segment model for myocardial segmentation. [37] Shading indicates percentage of native T1 values in that segment that were abnormal (> 1050). In basal slices, ROIs were generated on two septal segments each (segment 2 and 3) and a lateral wall region (combined segments 5 and 6). In mid slices, ROIs were generated on two septal segments each (segment 8 and 9) and a lateral wall region (combined segments 11 and 12). In apical slices, ROIs were generated on the septal (segment 14) and lateral (segment 16) segments. Of note, per lab standard, anterior and inferior segments were not analyzed to avoid partial volume effect

Native T1 parametric maps were deidentified and analyzed using additional offline software (OsiriX, Bernex, Switzerland). The middle 6 slices were used (2 basal, 2 mid, and 2 apical slices), to minimize through-plane motion artifacts associated with the most basal and apical slices. Based on the American Heart Association 17-segment model for myocardial segmentation [37], 16 total regions of interest (ROIs) were generated, as demonstrated in Fig. 1. Per lab standard [26], ROIs were traced in the septal and lateral walls only to avoid partial volume effect with fat and lungs in the anterior wall and partial volume effect with the diaphragm and stomach in the inferior wall commonly found in the pediatric population. ROIs were traced by a blinded reviewer using the “middle-third” technique to avoid artifacts and blood pool at the endocardial border, per lab standard [26, 38], yielding 16 segmental average regional voxel T1 values. Global mean and peak native T1 values were quantified using these 16 segmental T1 values.

A second reviewer traced ROIs on 20% of cases (10 cases) to measure interobserver variability.

Given the myriad of factors contribution to local variation in T1 values, our institution created and maintains a local normal T1 database [16], which has both general norms (900–1050 ms) and normal values per body surface area (BSA) quartile. Per internal local studies on healthy control patients, normal T1 values were considered 900–1050 ms, with values above 1050 ms considered to be abnormal (Additional file 1: Table S2).

### Texture analysis

**Fig. 2:**
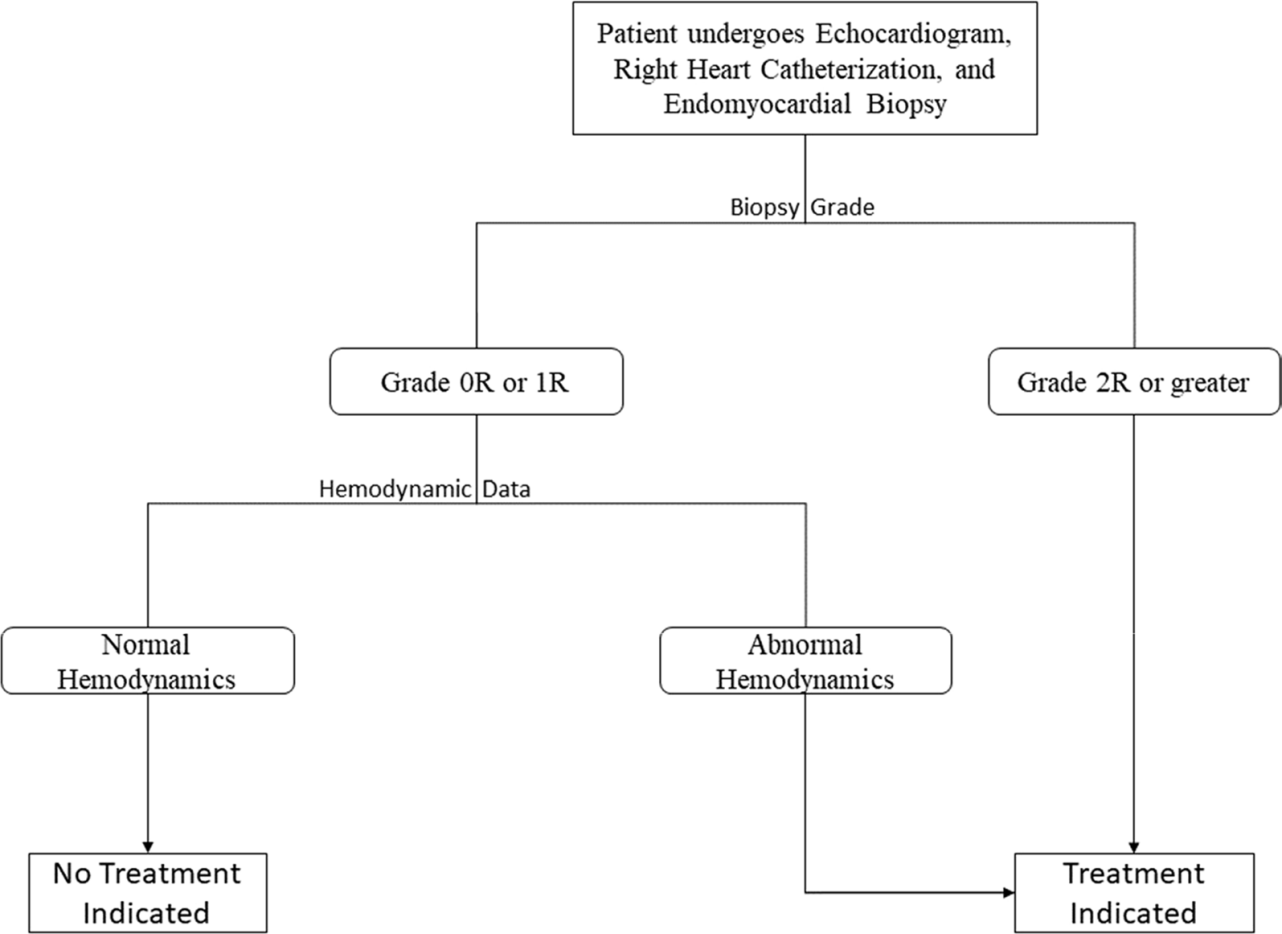
Demonstrated is the clinical treatment algorithm at our institution. Patients undergo transthoracic echocardiogram, right heart catheterization, and endomyocardial biopsy at each surveillance encounter. Coronary angiography is performed if indicated. Of note, the 50 cases included this study also underwent CMR per research protocol and right heart catheterization was performed under CMR guidance

LV myocardium was semiautomatically segmented using Otsu thresholding from T1 maps with offline software (Seg3D, Scientific Computing and Imaging Institute, Salt Lake City, Utah, USA). Blood pool and epicardial myocardium were carefully excluded. Image texture analysis (Fig. 2) was performed on a single mid-ventricular slice, using a previously-described texture toolbox [39] in MATLAB (The MathWorks Inc., Natick, Massachusetts, USA). Segmented maps were normalized to 256 intensity levels. A 256 × 256 Gy-level co-occurrence matrix (GLCM) was created, quantifying the frequency with which a pixel of a given intensity neighbors a pixel of another given pixel intensity. The GLCM is constructed such that each entry (*i, j*) in the GLCM quantifies the number of times that a pixel of intensity *i* neighbors a pixel of intensity *j*. An image with more homogeneous pixel intensity is thus more diagonally dominant than one with pixel heterogeneity. To quantify image heterogeneity, nine GLCM-based texture features were computed for each slice as previously described: [30, 40–42] energy, contrast, entropy, homogeneity, correlation, sum average, variance, dissimilarity, and autocorrelation (Additional file 1: Table S1).

### EMB fibrosis percentage analysis

EMB specimens underwent staining with Masson’s trichrome following a standard laboratory staining protocol. Whole slide scanning was performed using Tissue Scope (Huron Technologies, Ontario, Canada). Using ImageScope (Aperio, Vista, California, USA), endocardial collagen and artifacts were manually excluded. A pixel counting algorithm was developed by setting a colorimetric threshold specific to blue stained collagen. Using this algorithm, percentage tissue fibrosis was calculated per biopsy. Surgical pathologists served as expert readers, with involvement in the creation and tuning of the percentage fibrosis tool.

### Statistical analysis

Interclass correlation coefficient was used to verify interobserver variability. Student’s t-test was performed on native T1 values and parametric map texture features of cases with and without rejection, as defined by clinical rejection (Group A vs Groups B + C above) and by biopsy grade (Grade 0R vs Grade 1R or greater); ANOVA analysis was also performed across the three treatment groups. A receiver operating characteristic curve was created for mean T1 values, peak T1 values, and significant texture analysis features. Pearson correlation was performed on T1 mean and peak values against markers of graft dysfunction, which included serum BNP and hemodynamic data from echocardiography, catheterization, and CMR. All statistical analyses were performed using GraphPad Prism version 9.0.0 for Windows (GraphPad Software, San Diego, California, USA).

## Results

### Patient cohort

Twenty-four pediatric patients in 50 cases (12.2 ± 4.6 years, graft age 5.3 ± 4.1 years, 36% female) underwent study procedures for surveillance (70%), suspected rejection (4%), and follow-up of prior rejection (26%). Thirty-seven cases (74%) were in Group A (no rejection, no therapy changes), 6 cases (12%) in Group B (mild rejection, minor therapy changes), and 7 cases (14%) in Group C (rejection present, major therapy changes). For analysis purposes, groups B + C represented those cases with clinical rejection. There was no difference in mean age, graft age, or graft ischemia time between Group A and Group B + C (Table 1). LV ejection fraction on echocardiogram was lower in Group B + C than Group A (60.8 ± 3.4%vs 64.3 ± 5.2%, p = 0.009), though both were normal (Table 2). A higher percentage of cases in Group B + C, compared to Group A, were positive for DSAs (53.8% vs 20%, p = 0.051).

**Table 1:** Patient cohort: demographics

Demographic	All cases	Group A	Groups B and C	P value
All cases	n = 50	n = 37 (74%)	n = 13 (26%)	
Male	64.0%	67.6%	53.8%	0.412
White	77.3%	75.8%	81.8%	0.915
Hispanic	32.0%	24.3%	53.8%	0.083
Mean age (years)	12.2 ± 4.6	11.9 ± 4.8	13.3 ± 4.2	0.326
Mean graft age (years)	5.3 ± 4.1	5.4 ± 3.7	5.1 ± 5.2	0.882
Graft ischemia time (minutes)	228 ± 44	225 ± 43	236 ± 49	0.531
Coronary vasculopathy	0%	0%	0%	1.000
Clinical concerns for rejection	4.0%	2.7%	7.7%	0.549
Indication for catheterization: surveillance	70%	75.7%	53.8%	0.191
Number of prior rejection episodes	6.3 ± 6.1	5.8 ± 6.1	7.8 ± 6.1	0.315
NT-pro BNP Level (pg/mL)	787 ± 1477	332 ± 530	1663 ± 2549	0.099
Positive for donor-specific antibodies	30.2%	20.0%	53.8%	0.051
Echo LV ejection fraction (%)	63.4 ± 5.0	64.3 ± 5.2	60.8 ± 3.4	0.009
Cath RA mean pressure (mmHg)	10 ± 4	9 ± 3	12 ± 5	0.096
Cath RV systolic (mmHg)	28 ± 5	28 ± 5	28 ± 5	0.954
Cath RVEDP (mmHg)	11 ± 4	11 ± 4	13 ± 6	0.137
Cath average RPCW/LPCW (mmHg)	12 ± 5	11 ± 3	15 ± 6	0.071
Biopsy grade > 0R	34%	16.2%	84.6%	0.001
Biopsy grade > 1R	4%	0%	15.4%	0.014

*NT-Pro BNP* N-terminal pro-hormone brain natriuretic peptide, *LV* left ventricular, *RV* right ventricular, *RVEDP* right ventricular end-diastolic pressure

**Table 2:** Indication for treatment

Group	Biopsy grade	Treatment received	Reason for treatment
B	Grade 1R	Tacrolimus goal increased	Histological: Persistent 1R biopsy × 3
B	Grade 1R	Tacrolimus goal increased	Hemodynamics: systolic dysfunction (mildly decreased left ventricular function)
B	Grade 0	Low dose steroids	Hemodynamics: diastolic dysfunction (elevated right end diastolic pressure and capillary wedge pressure); new positive DSA
B	Grade 1R	Tacrolimus goal increased	Hemodynamics: systolic dysfunction (mildly decreased left ventricular function)
B	Grade 1R	Tacrolimus goal increased	Hemodynamics: diastolic dysfunction (elevated pulmonary wedge pressures)
B	Grade 1R	Oral steroids (not pulse dose)	Hemodynamics: diastolic dysfunction (elevated right end diastolic pressure and pulmonary capillary wedge pressure)
B	Grade 0	MMF dose increased	Hemodynamics: systolic dysfunction (mildly decreased biventricular function)
C	Grade 1R	Oral pulse steroids	Hemodynamics: diastolic dysfunction (elevated right end diastolic pressures and capillary wedge pressure)
C	Grade 1R	IVIG and rituximab	Hemodynamics: systolic dysfunction (decreased biventricular function, requiring milrinone)
C	Grade 1R	IV pulse steroids	Hemodynamics: diastolic dysfunction (elevated right end diastolic pressures and pulmonary capillary wedge pressure) and decreased cardiac index (requiring epinephrine)
C	Grade 1R	IV pulse steroids, IVIG, rituximab	Hemodynamics: diastolic dysfunction (elevated right end diastolic pressure and capillary wedge pressure); Worsening DSA
C	Grade 2R	IV pulse steroids, thymoglobulin	Histological and hemodynamics: diastolic dysfunction (elevated right end diastolic pressures and right atrial pressure)
C	Grade 2R	IV pulse steroids, thymoglobulin	Histological

*DSA* donor specific antibodies, *IV* intravenous, *IVIG* intravenous immunoglobulin

Similarly, a higher percentage of cases in Group B + C had biopsies with cellular rejection grade > 0R compared to Group A (84.6% vs 16.2%, p = 0.001).

Two patients in Group B + C who had Grade 0R biopsies and abnormal hemodynamics, prompted modification of immunosuppressive therapy (Table 2). Of the six cases in Group A with Grade 1R biopsies, all had normal hemodynamics on echocardiogram, catheterization, and CMR; further all six cases were receiving more frequent surveillance due to history of rejection and continued to show improvement during these cases. There were no cases of antibody mediated rejection among the 50 cases.

### T1 parametric map analysis

Intraclass correlation coefficient of 20% of cases, with regions of interest traced by 2 reviewers, was 0.829 for global mean T1 and 0.830 for peak T1.

### T1 correlation with clinical rejection

**Fig. 3:**
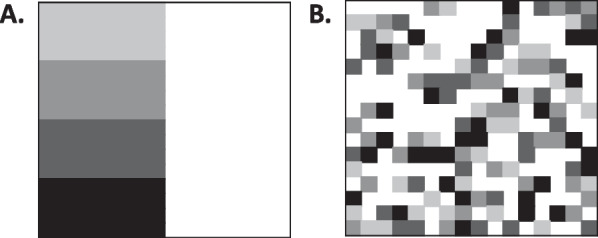
Representation of texture analysis of two example images that have the same pixel intensity mean, standard deviation, and distribution but differ in texture features. Image** A** has higher energy with lower entropy and variance than Image** B**

**Fig. 4:**
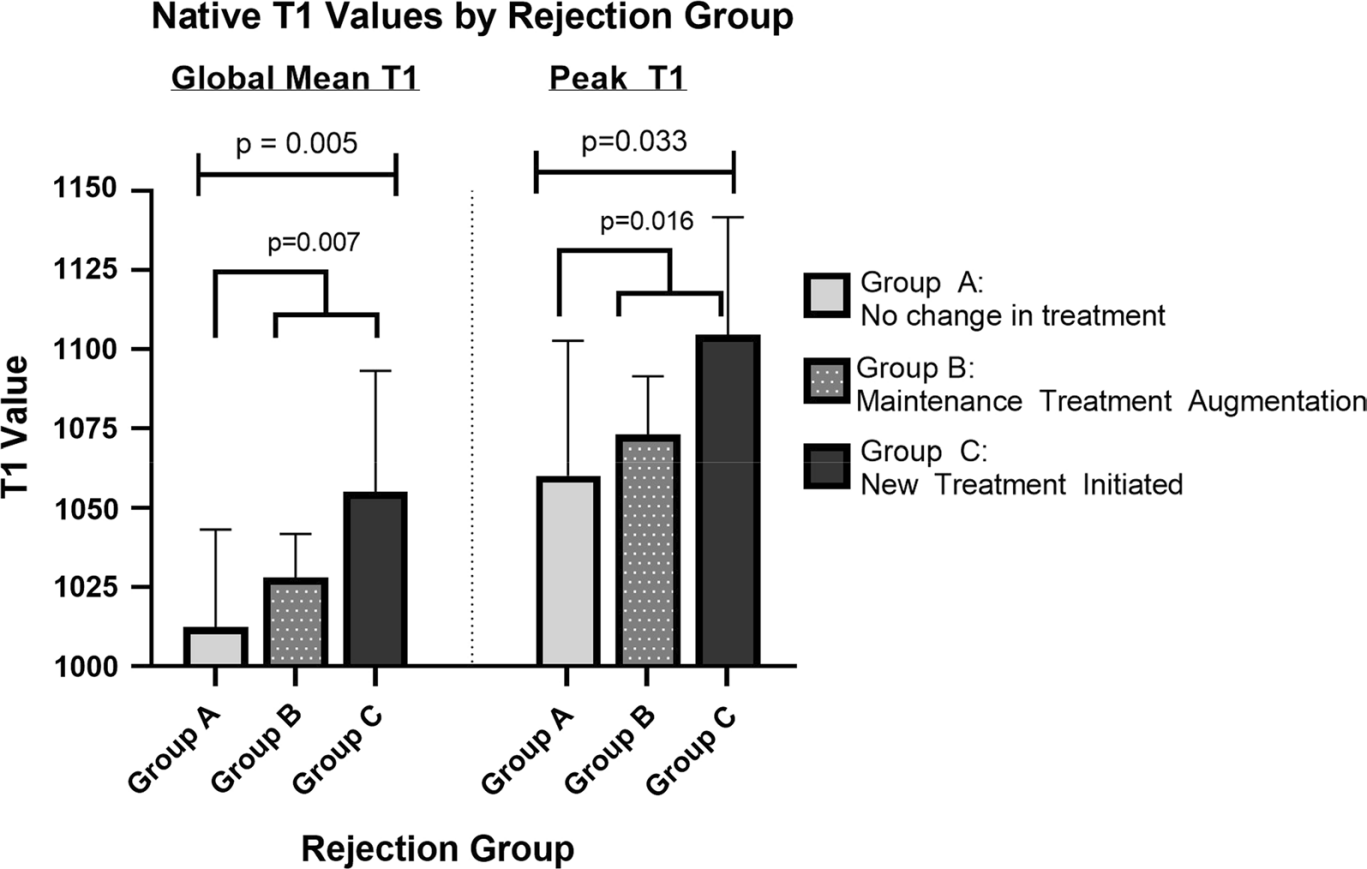
ANOVA analysis between three groups of global mean T1 and peak T1 values yielded p value of 0.0005 and 0.033 respectively. T-test analysis between group A (no change in treatment) versus combined groups B (maintenance treatment augmentation) and C (new treatment initiated) of global mean T1 and peak T2 values yielded pvalue of 0.007 and 0.016 respectively

**Fig. 5:**
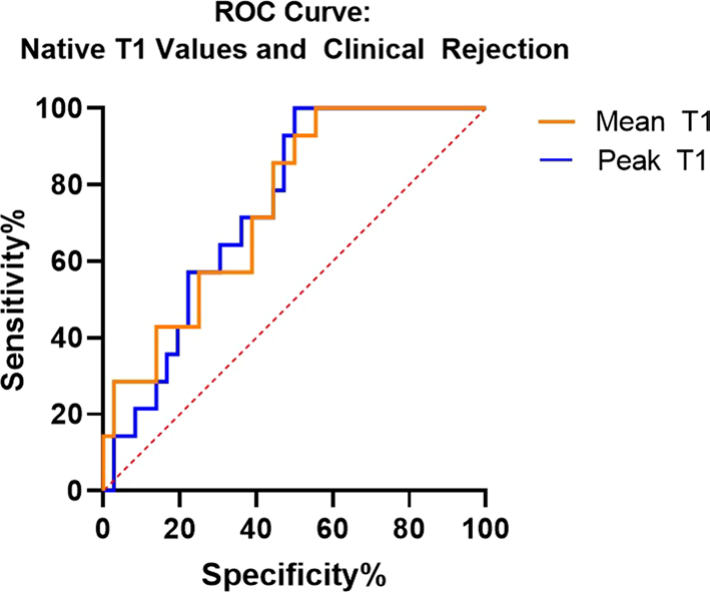
Receive Operative Characteristics Curve demonstrating sensitivity and specificity of mean and peak T1 values between groups with and without clinical rejection requiring treatment. AUC for mean T1 values was 0.746 (p = 0.007) and for peak T1 values was 0.730 (p = 0.012)

A monotonic, increasing trend was noted in both mean and peak T1 values, with increasing degree of rejection (Figs. 3, 4), with peak T1 values in the abnormal range (T1 values greater than 1050) for Group B and C. Area under the curve (AUC) of receiver operating characteristic curve of mean T1 values was 0.746 (p = 0.007) and of peak T1 values was 0.730 (p = 0.012) (Fig. 5). Notably, ROC analysis demonstrated 100% sensitivity at peak T1 values > 1050 ms, which is the cutoff of normal vs abnormal T1 values at our institution based on internal studies.

Interestingly, T1 values did not differ between cases with Grade 0R and those with Grade 1R or higher cellular rejection [mean T1: 1018 ± 28 ms vs 1029 ± 45 ms respectively (p = 0.388); peak T1: 1067 ± 44 ms vs 1075 ± 44 ms respectively (p = 0.547)]. Of note, 2 of 13 cases (15%) in group B + C had biopsies with grade 0R rejection, were treated for clinical rejection, and both were found to have elevated peak T1 values.

### T1 correlation with graft function

Mean T1 values demonstrated moderate correlation with BNP (r = 0.59) and mean pulmonary artery pressure (r = 0.33). Peak T1 values demonstrated moderate correlation with BNP (r = 0.52), right atrial pressure (r = 0.40), mean pulmonary artery pressure (r = 0.46), RVEDP (r = 0.36), and average pulmonary capillary wedge pressure (r = 0.33). T1 values did not correlate with other hemodynamic markers (Table 3). When cases with clinical rejection were removed, leaving only those cases with possible graft dysfunction, peak T1 correlated with BNP moderately (r = 0.54) but with no other markers.

**Table 3:** Correlation of native T1 values and markers of graft dysfunction

Marker	Coefficient values (r)			
	vs Mean T1	p-value	vs Peak T1	p-value
BNP	0.59*	< 0.0001	0.52*	< 0.001
Left ventricular ejection fraction	− 0.20	0.186	− 0.33*	0.024
Average mitral E/e’	− 0.15	0.334	− 0.05	0.755
RA mean pressure	0.23	0.138	0.40*	0.008
RV systolic pressure	− 0.04	0.810	0.08	0.603
RVEDP	0.18	0.239	0.36*	0.017
Main pulmonary artery mean pressure	0.33*	0.043	0.46*	0.005
Avg pulmonary capillary wedge pressure	0.24	0.125	0.33*	0.034
LVEDV	− 0.08	0.584	− 0.22	0.130
RVEDV	− 0.11	0.466	− 0.21	0.151

*LVEDV* left ventricular end-diastolic volume, *RA* right atrial, *RV* right ventricular, *RVEDP* right ventricular end-diastolic pressure, *RVEDV* right ventricular end-diastolic volume

*Significant p-value

### T1 correlation with fibrosis percentage

Fibrosis percentage, calculated from random EMB of the RV, did not demonstrate a difference between cases of clinical rejection (Group B + C), versus cases without rejection (Group A) (8.2% ± 3.1 vs 7.4% ± 5.2, p = 0.703). Fibrosis percentage did not correlate with hemodynamic markers listed above or with mean or peak T1 values.

### Native T1 texture analysis

**Fig. 6:**
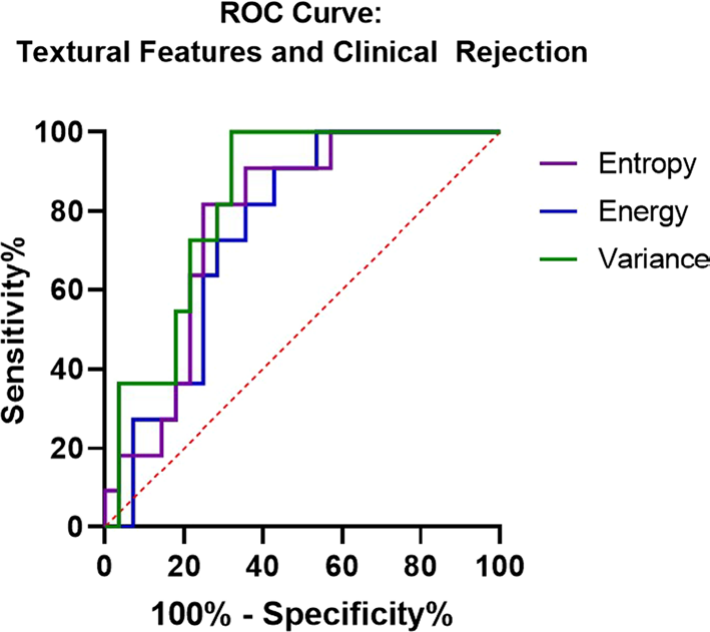
Receive Operative Characteristics Curve demonstrating sensitivity and specificity of 3 textural features noted to show significant differences between groups with and without clinical rejection requiring treatment. Noted is an AUC of 0.750 (p = 0.016) for energy, 0.779 (p = 0.007) for entropy, and 0.831 (p = 0.002) for variance

Image texture analysis demonstrated that mid-ventricular short axis slices from Group A differed from groups B and C in three of nine texture features computed. Energy, a measure of image homogeneity, was higher in Group A than Groups B + C (p = 0.033). Entropy and variance, which reflect randomness and heterogeneity, were higher in Groups B + C than in Group A (p = 0.008 and p = 0.001 respectively). ROC analysis of these texture features demonstrated AUC of 0.750 (p = 0.016) for energy, 0.779 (p = 0.007) for entropy, and 0.831 (p = 0.002) for variance (Fig. 6). Other texture features did not differ between the groups.

## Discussion

This prospective study demonstrates that mean and peak native T1 values correlate with both degree of clinical rejection and graft dysfunction in pediatric heart transplant patients. Further, no correlation between native T1 values and biopsy findings was demonstrated, indicating that native T1 mapping holds promise to identify clinically important changes in the heart transplant population, possibly outperforming random EMB of the RV. CMR is emerging as a useful tool for rejection screening in pediatric transplant patients, particularly when biopsy negative clinical rejection is present. In addition, texture analysis of parametric T1 maps identified additional promising trends for detection of rejection using imaging heterogeneity. Similar utility of native T1 values has been noted in adult studies, which demonstrated T1 and T2 mapping as useful tools for detecting rejection and therefore reducing the need for invasive EMB. Specifically, Imran et al. demonstrated an excellent negative predictive value of T1 mapping for cardiac allograft rejection [20]. Similarly, Vermes et al., Dolan et al., and Usman et al., have demonstrated higher T2 levels and higher extracellular volume fraction (ECV) in patients with acute rejection [18, 19, 43]. Pediatric studies have noted higher native T1 values in all pediatric heart transplant patients compared to controls without transplants [44] and higher native T2 values in cases of allograft rejection [38]. However, in other studies in which rejection was defined solely by biopsy grade, no difference in T1 values was demonstrated [45]. Using a larger population and whole-heart imaging, we have demonstrated a difference in T1 values between cases of rejection and cases without rejection, when rejection is defined clinically by necessity of treatment rather than exclusively by biopsy grade. Further, due to significant inter-center variability in the frequency of EMB for rejection surveillance, prior studies have investigated the yield of EMB and its impact on outcomes in the pediatric population [46, 47]. Studies investigating frequency of EMB both in the first year post-transplantation [47] when rejection risk is highest as well as long-term routine surveillance [46], demonstrated similar outcomes between high intensity and low intensity protocols. Adult studies have supported discontinuing routine EMB for long-term surveillance [48, 49].

Though texture analysis has been demonstrated in other pediatric conditions such as myocarditis [32, 33] and cardiomyopathies [34, 35], it has not previously been applied in pediatric heart transplant population and we therefore sought to do so. Texture analysis allows for the evaluation of heterogeneity in relative voxel intensities rather than using absolute voxel intensities [30, 31], offering the advantage of overcoming lack of normal T1 values between institutions given differences in field strengths, vendors, and individual scanners [16].

Diastolic dysfunction impacts prognosis of various etiologies of chronic heart disease [50], including graft failure [51]. Several studies have demonstrated a relationship between diastolic dysfunction and myocardial fibrosis as measured by histological analysis [15, 51] and late post-gadolinium myocardial enhancement CMR [50]. In our study, random EMB fibrosis percentage did not correlate with T1 values or with markers of graft dysfunction. Further, fibrosis analysis of random EMB also did not differ between cases of clinical rejection. It is unclear if this reflects a limitation in the ability of T1 values to assess for myocardial fibrosis burden; however, it is more likely that fibrosis percentage measurements from randomly sampled EMB tissue samples do not accurately reflect whole heart myocardial fibrosis burden. Non-invasive markers, such as BNP and echocardiography mitral E/e’, have also been shown to correlate with RVEDP and pulmonary capillary wedge pressure [52], markers of graft dysfunction. Though native T1 values showed moderate correlation with hemodynamic markers and BNP when patients in acute clinical rejection were included, this correlation was not found when looking only at patients without rejection. Perhaps, native T1 values, while showing promise in identifying clinical rejection and in assessing graft dysfunction in patients with rejection, may be limited in their ability to assess for more chronic graft changes that occur, including those mediated by fibrosis.

### Limitations

There are limitations to this work; this is a single center study including a relatively small cohort which includes longitudinal, repeat encounters. We recognize that though this is a prospective study, the definition of clinical rejection is retrospectively based on decision to treat. However, these treatment groups are in line with the clinical treatment algorithm at our institution and therefore we find are a reliable outcome measure to compare. Also of note is that only septal and lateral segments were used for T1 value measurements. Given the nature of T1 mapping, we preferred to analyze only highly reliable data at the cost of excluding certain segments. In the pediatric population, particularly with patients as small as 15 kg, the anterior and inferior segments are subject to partial volume effect from epicardial fat and lungs for anterior segments and diaphragm and stomach for inferior segments, leading to unreliable data. Therefore, these segments were not included in the analysis, as is consistent with lab standards [26]. It is also important to note that very few patients underwent this procedure due to clinical concern for rejection, but rather for screening. Further, screening protocols and treatment protocols differ between institutions as standardized guidelines do not exist; therefore, further investigation at a multi-institutional level is required.

Despite these limitations, we find it promising that CMR serves as a noninvasive screening tool during surveillance encounters and may be used to identify those patients that may be at higher risk of rejection and therefore require further evaluation. Transplant rejection surveillance remains a multi-faceted approach, including assessment of clinical presentation, echocardiography, catheterization hemodynamics, and serum markers. Our team sought to demonstrate the possibility of a synergistic value of a combined CMR and EMB protocol for evaluating patients. In our population, two patients had Grade 0R biopsies but required treatment due to abnormal hemodynamics; these same patients demonstrated peak T1 values > 1050 ms, considered abnormal in per institutional normal values, further emphasizing the utility of CMR. ROC analysis demonstrates 100% sensitivity at peak T1 values > 1050 ms, demonstrating the possibility of a model in which patients requiring transplant rejection surveillance undergo CMR including parametric mapping, right heart catheterization, and hemodynamic measurements as initial screening. For those patients with abnormal findings, EMB would be performed and also inform treatment decision. In our analysis, if this model were used, all patients who required treatment would have underwent EMB and the appropriate treatment. Of the 37 patients who did not require treatment, 15 patients would have been saved from invasive EMB. Further, at our institution where CMR guided right heart catheterization is available and routinely performed for hemodynamic data, these patients are exposed to radiation only during coronary angiography, typically only performed at annual surveillance encounters. CMR, with its ability to perform radiation free evaluations that allow for hemodynamic assessment, particularly with the use of CMR guided right heart catheterization, and for entire myocardium assessment for fibrosis and edema, may be a promising tool in the pediatric heart transplant population. Further investigation is needed in the other ways in which CMR may serve as a useful tool in transplant screening, including the potential for guiding EMB. Preliminary work completed by our group shows parametric mapping patterns of T1/T2 elevations, or hotspots [53], in myocardial diseases such as rejection, reinforcing that these pathological changes are not uniform in nature. This has been suspected in the past given the false negative rate of random EMB [4–6]. Evaluation of these hotspots using guided EMB may provide further insight.

## Conclusion

CMR native T1 parametric mapping demonstrates utility in identifying rejection and assessing for graft dysfunction in pediatric heart transplant patients, possibly beyond random endomyocardial biopsy. Further work is needed to determine how CMR can best fit into the current clinical multi-faceted approach to transplant rejection.

## Supplementary Information

**Additional file 1: Table S1.** Definition and calculations of gray-level co-occurrence matrix texture features [30, 40–42].

## Data Availability

The datasets used and/or analyzed during the current study are available from the corresponding author on reasonable request.

## Abbreviations

BNP: Brain natriuretic peptide
BSA: Body surface area
CMR: Cardiovascular magnetic resonance
DSA: Donor specific antibodies
ECV: Extracellular volume fraction
EMB: Endomyocardial biopsy
GLCM: Gray-level co-occurrence matrix
IRB: Institutional review board
ISHLT: International society of heart and lung transplantation
IV: Intravenous
IVIG: Intravenous immunoglobulin
LV: Left ventricle/left ventricular
MOLLI: Modified look-locker inversion
RA: Right atrial
ROI: Region of interest
RV: Right ventricle/right ventricular
RVEDP: Right ventricle end diastolic pressure
