# Supplementary material for: Native T1 mapping detects both acute clinical rejection and graft dysfunction in pediatric heart transplant patients
Source: J Cardiovasc Magn Reson. 2022 Oct 3;24:51. doi: 10.1186/s12968-022-00875-z (PMC9531384; doi:10.1186/s12968-022-00875-z)
Supplement: Supplementary file 1 — Additional file 1: Table S1. Definition and calculations of gray-level co-occurrence matrix texture features [30, 40–42]. [file 12968_2022_875_MOESM1_ESM.docx]

**Supplemental Material**

**Table S1:** Definition and calculations of gray-level co-occurrence matrix texture features^30,40–42^

|  | **Feature** | **Definition** | **Equation*** |
| --- | --- | --- | --- |
|  | Energy | overall image heterogeneity | $\sum_{i=1}^{N_{g}} \sum_{j=1}^{N_{g}} \left[ p\left( i,j \right) \right]^{2}$ |
|  | Contrast | local gray level variation | $\sum_{i=1}^{N_{g}} \sum_{j=1}^{N_{g}} \left( i-j \right)^{2}p\left( i,j \right)$ |
|  | Entropy | randomness of gray level distribution | $-\sum_{i=1}^{N_{g}} \sum_{j=1}^{N_{g}} p\left( i,j \right)\log_{2} \left( p\left( i,j \right) \right)$ |
|  | Homogeneity | gray level similarity of neighboring voxels | $\sum_{i=1}^{N_{g}} \sum_{j=1}^{N_{g}} \frac{p\left( i,j \right)}{1+\left\vert i-j \right\vert}$ |
|  | Correlation | linear dependence of gray level on neighboring voxels | $\sum_{i=1}^{N_{g}} \sum_{j=1}^{N_{g}} \frac{\left( i-\mu_{i} \right)\left( j-\mu_{j} \right)p\left( i,j \right)}{\sigma_{i}\sigma_{j}}$ |
|  | Sum Average | relationship between lower and higher intensity voxel pairs | $\frac{1}{N_{g}N_{g}}\sum_{i=1}^{N_{g}} \sum_{j=1}^{N_{g}} \left[ ip\left( i,j \right)+jp\left( i,j \right) \right]$ |
|  | Variance | heterogeneity weighted toward voxel pairs with higher deviation from mean | $\frac{1}{N_{g}N_{g}}\sum_{i=1}^{N_{g}} \sum_{j=1}^{N_{g}} \left[ \left( i-\mu_{i} \right)^{2}p\left( i,j \right)+\left( j-\mu_{j} \right)^{2}p\left( i,j \right) \right]$ |
|  | Dissimilarity | absolute intensity difference between neighboring voxels | $\sum_{i=1}^{N_{g}} \sum_{j=1}^{N_{g}} \left\vert i-j \right\vert p\left( i,j \right)$ |
|  | Autocorrelation | fineness or coarseness of texture | $\sum_{i=1}^{N_{g}} \sum_{j=1}^{N_{g}} ijp\left( i,j \right)$ |

**a.** *P* defines the GLCM, and *p(i,j)* represents the number of times voxels of gray-level *i* were neighbors with voxels of gray-level *j* ; *N_g_* represents the number of pre-defined gray levels;
**b.** where:

**Table S2:** Normal T1 ranges based on BSA

| Normal Ranges for T1 Septal based on BSA |  |  |
| --- | --- | --- |
| BSA Quartile | Mean T1 Septal | 95% CI Normal Range |
| Quartile 1 (0.70 to < 1.35) | 1019.1 | (933.1, 1105.1) |
| Quartile 2 (1.35 to < 1.58) | 1012.5 | (926.4, 1098.5) |
| Quartile 3 (1.58 to < 1.85) | 1002.5 | (916.2, 1088.9) |
| Quartile 4 (1.85 to 2.17) | 979.1 | (893.1, 1065.1) |
|  |  |  |
|  |  |  |
| Normal Ranges for T1 Lateral based on BSA |  |  |
| BSA Quartile | Mean T1 Septal | 95% CI Normal Range |
| Quartile 1 (0.70 to < 1.35) | 1014.2 | (920.4, 1107.9) |
| Quartile 2 (1.35 to < 1.58) | 975.3 | (881.6, 1069) |
| Quartile 3 (1.58 to < 1.85) | 969.4 | (875.4, 1063.5) |
| Quartile 4 (1.85 to 2.17) | 953.9 | (860.2, 1047.7) |
|  |  |  |
|  |  |  |
|  |  |  |
